# Mitochondrial Proteomics of Antimony and Miltefosine Resistant *Leishmania infantum*

**DOI:** 10.3390/proteomes3040328

**Published:** 2015-10-21

**Authors:** Isabel M. Vincent, Gina Racine, Danielle Légaré, Marc Ouellette

**Affiliations:** Centre de Recherche en Infectiologie du CHU de Québec, Université Laval, Québec City, QC G1V 4G2, Canada; E-Mails: Isabel.vincent@glasgow.ac.uk (I.M.V.); Gina.Racine@crchudequebec.ulaval.ca (G.R.); daniellelegare1@gmail.com (D.L.)

**Keywords:** *Leishmania*, mitochondrion, proteome, antimony, miltefosine

## Abstract

Antimony (SbIII) and miltefosine (MIL) are important drugs for the treatment of *Leishmania* parasite infections. The mitochondrion is likely to play a central role in SbIII and MIL induced cell death in this parasite. Enriched mitochondrial samples from *Leishmania* promastigotes selected step by step for *in vitro* resistance to SbIII and MIL were subjected to differential proteomic analysis. A shared decrease in both mutants in the levels of pyruvate dehydrogenase, dihydrolipoamide dehydrogenase, and isocitrate dehydrogenase was observed, as well as a differential abundance in two calcium-binding proteins and the unique dynamin-1-like protein of the parasite. Both mutants presented a shared increase in the succinyl-CoA:3-ketoacid-coenzyme A transferase and the abundance of numerous hypothetical proteins was also altered in both mutants. In general, the proteomic changes observed in the MIL mutant were less pronounced than in the SbIII mutant, probably due to the early appearance of a mutation in the miltefosine transporter abrogating the need for a strong mitochondrial adaptation. This study is the first analysis of the *Leishmania* mitochondrial proteome and offers powerful insights into the adaptations to this organelle during SbIII and MIL drug resistance.

## 1. Introduction

The *Leishmania spp* genus of protozoan parasites contains more than 20 species that are responsible for several maladies termed leishmaniasis. The parasites infect an estimated 12 million people in Asia, Europe, the Middle East, Africa, and South America [1]. Since their discovery in the 1940s, the toxic parenteral pentavalent antimony (Sb^V^) compounds have been the mainstay of treatment for all types of leishmaniasis and are still the first-line treatment in most areas, although clinical resistance is often observed. As the incidence of resistance increases, notably in the Bihar state of Northern India, the dose of drug recommended for use in patients has been increasing in addition to the length of treatment. However, as antimonials already display unacceptable levels of toxicity, the dose cannot be increased further and other ways to make this class of drugs more effective are under investigation. Resistant strains have been widely studied in the laboratory in an attempt to elucidate the drug’s mechanisms of resistance, with many resistance-associated genes identified [2]. SbIII (antimony in its 3^+^ oxidation state) is sequestered in *Leishmania* conjugated to trypanothione (TSH) or glutathione (GSH) [3,4,5] through an ABC transporter termed MRPA [6], which correlates with an increase in the production of thiols in resistant isolates [4,7,8]. The *MRPA* gene is often amplified in extrachromosomal circles of DNA and overexpressed upon selection of the parasite for SbIII resistance [9,10]. Host cells have also been shown to upregulate an analogue of MRPA when infected with antimony resistant *L. donovani* [11]. The increased thiol production in antimony resistant *Leishmania* [4] also results in a greater ability for the cells to cope with oxidative stresses [12]. The mechanism of action of antimony in *Leishmania* parasites is still unclear although it has been shown that the drug inhibits glycolysis and β-oxidation of fatty acids in these parasites. It has been also demonstrated that SbIII induces apoptotic-like features including accumulation of reactive oxygen species (ROS), a drop in mitochondrial membrane potential, genomic DNA degradation, and an increase in intracellular calcium [13]. 

Miltefosine (MIL), an alkylphosphocholine originally developed as an anticancer drug, has been used since 2005 in first line for the oral treatment of visceral leishmaniasis in the Indian subcontinent. Although clinical resistance is extremely rare, MIL resistance is easily induced in *in vitro* conditions. The main resistance mechanism is associated with failure of the MIL-dedicated transporter, the aminophospholipid translocase LdMT, to transport the drug [14]. A number of mutations in the translocase or in its beta subunit LdRos3 were found to confer MIL resistance [14,15,16]. Miltefosine modulates cell surface receptors, affecting inositol and phospholipase metabolism, signal transduction, and Ca^2+^ homeostasis [17,18,19].

An increasing body of evidence suggests that *Leishmania* undergoes cell death resembling apoptosis upon treatment with antimony or MIL, a process closely linked with mitochondria, the main source of ROS [20,21,22,23]. There is still considerable debate on whether cell death in *Leishmania* is regulated or incidental however [24], but since *Leishmania* cell death pathways differ from those of typical mammalian apoptosis, it remains an interesting subject of investigation. 

Mitochondria are the power houses of cells, producing much of the cell’s energy through the tricarboxylic acid (TCA) cycle and oxidative phosphorylation. Mitochondria are also involved in the parasite response to pharmacological perturbation, being the main source of ROS and controlling cell death. It has been shown that ROS are produced when *Leishmania* are treated with either MIL or antimony [12,13,25,26]. Parasites belonging to the Kinetoplastida eukaryotic branch are unusual in that each cell contains one, enlarged mitochondrion, stretching the majority of the length of the cell. A comprehensive analysis of the *Trypanosoma brucei* procyclic form (a related kinetoplastid parasite) mitochondrial proteome has previously been published [27]. In this study, mitochondrial vesicles were enriched on a Percoll gradient and proteins were designated as being mitochondrial when they were enhanced in the mitochondria-enriched sample and had a predicted mitochondrial location (predicted using MitoProt/Signal P) [27]. This analysis identified many more proteins than were previously thought to be located in the mitochondrion, indicating that there may be many processes occurring in this enlarged organelle of which we have limited knowledge. We also tested whether miltefosine and antimony resistance induced any changes to the mitochondrial proteome of *Leishmania* and whether some of these changes were common in antimony and miltefosine resistant mutants.

## 2. Experimental Section

### 2.1. Reagents

All reagents were acquired from Sigma Aldrich (St-Louis, MO, USA) or Fluka (St-Louis, MO, USA), unless otherwise stated. 

### 2.2. Culture and Resistance Selection

The *L. infantum* JPCM5 (MCAN/ES/98/LLM-877) promastigote cell line was used to select the derived lines MF200 and SbIII2000.2 by a stepwise increase in drug pressure and these two resistant lines were described previously [19]. These lines were resistant to 200 µM miltefosine (Cayman Chemical, Ann Harbor, MI, USA) or 2 mM SbIII respectively and grew slightly more slowly than wild-type (Supplementary File 1, Figure S1). Cells were maintained in medium 199 (Gibco/Thermo Fisher, Waltham, MA, USA) supplemented with the appropriate drug as well as with 10% heat-inactivated foetal bovine serum and 10 µg/mL haemin at 25 °C. 

IC_50_s were taken by serial dilution of drug (from a starting concentration of 2 mM for SbIII and 400 µM for MIL) in transparent 96-well plates before addition of logarithmic stage cells at a final density of 2.5 × 10^6^/mL and incubated at 28 °C, shaking for 72 h. The optical density of each well in the plate was read at λ = 600 nm and analysed with Graphpad Prism (GraphPad Software, La Jolla, CA, USA, version 5) using non-linear regression analysis. All IC_50_s were taken in at least triplicate.

### 2.3. Extraction and Purification of Mitochondria

Protocols for the extraction of mitochondria were adapted from methods by Hauser *et al* and Horváth *et al.* [28,29] (a summarised image of the procedure is illustrated in Supplementary File 1, Figure S2). Briefly, 800 mL of logarithmic phase cell culture in M199 (at 5 × 10^6^/mL) was washed in HEPES-NaCl and re-suspended to 2 × 10^9^/mL in SoTE buffer (20 mM Tris-HCl, 0.6 M sorbitol, 2 mM EDTA pH 7.8). Cells were lysed under 70 bar argon pressure for one hour followed by hypotonic lysis of nuclei in cold SoTE with 6 mM MgCl_2_ and 50 mg/mL DNAse I (Roche, Mississauga, ON, Canada) using a 25 G needle. DNA digestion was arrested after 30 min with 6 mM EDTA and intact organelles were washed in SoTE before further purification of mitochondria on a Nycodenz AG (Cedarlane Laboratories Ltd., Burlington, ON, Canada) gradient (50%:32%:28%:25%:21% *w*/*v* in SoTE). The 25%:28% interface had the greatest concentration of mitochondria. Mitochondria were quantified using the 2D Quant kit (GE Healthcare, Mississauga, ON, Canada) then stored in SoTE with 50% glycerol at −80 °C until ready to use in acrylamide gels. Four replicates were taken for each condition.

### 2.4. Protein Extraction

Soluble proteins were extracted from mitochondrial fractions using T8 buffer (7 M urea, 2 M thiourea, 3% (*w*/*v*) chaps, 20 mM DTT, 5 mM TCEP, 0.5% IPG pH 4–7 (GE Healthcare), 0.25% IPG pH 3–10 (GE Healthcare) with protease inhibitor cocktail and 50 mM Tris-HCl. Proteins were precipitated using a 2D clean-up kit (GE Healthcare) and re-suspended in T8 buffer. Protein concentrations were determined using the 2D Quant kit (GE Healthcare).

### 2.5. Western Blot 

SDS-PAGE was performed on a 12% acrylamide gel according to standard procedures. The BioRad Kaleidoscope ladder was used and the gel was stained in SYPRO Ruby (Life technologies, Carlsbad, CA, USA). The gel was transferred onto 0.2 µM nitrocellulose membrane (BioRad, Hercules, CA, USA), blocked in 5% milk (*w*/*v*) in TBS + 0.2% tween and probed with 1/1000 rabbit anti-histone 3 IgG, 1/4000 anti-HSP60 IgG (Assay Designs/Enzo Life Sciences, Farmingdale, NY, USA) or 1/5000 mouse anti-α-tubulin IgG for 90 min. Anti-rabbit IgG (GE Healthcare) was used to detect the anti-histone 3 antibody and anti-mouse IgG (Molecular Probes/Thermo Fisher, Waltham, MA, USA) was used to detect anti-α-tubulin and anti-HSP60. The expression was detected using the Immobilon western chemiluminescence kit (Millipore, Billerica, MA, USA). 

### 2.6. Sodium Dodecyl Sulfate (SDS)-PAGE 

Before performing 2D gel experiments, the WT mitochondrial protein extract was first evaluated by SDS-PAGE. Protein samples (30 µg) were mixed with 4× premixed protein sample buffer (BioRad) and β-mercaptoethanol (5% final concentration, Sigma Aldrich), and heated at 95 °C for 5 min. Protein mixtures were then loaded on Precast Criterion XT Bis-Tris gradient gels (4%–12% polyacrylamide, BioRad) and the SDS-PAGE separation was performed on a Criterion™ gel electrophoresis cell (BioRad) using a PowerPac 200 BioRad power supply set at 200 V for 50 min. For staining, gels were fixed in a solution of 40% methanol: 7% acetic acid for 1 h then incubated overnight with SYPRO Ruby Protein Gel stain (Life technologies). The destaining step was performed three times for 30 min each in a solution of 10% methanol: 7% acetic acid. Gel images were captured on a PerkinElmer ProExpress Proteomic Imaging system (PerkinElmer, Waltham, MA, USA). Each sample lane from the SDS-PAGE gels was cut in three fractions with disposable blade (MEE-1 × 5) mounted on a One Touch GridCutter (Gel Company Inc., San Francisco, CA, USA). Fractions were further broken into smaller gel pieces with scalpels then proteins were in-gel digested as described below.

### 2.7. Two Dimensional Protein Gels

2D gels were run in quadruplicate according to standard procedures, except for wild-type extracts, which were run in triplicate. pH 4–7 IPG buffer and bromophenol blue were added to 200 µg of protein and run on 24 cm pH 4–7 strips (GE Healthcare). Strips were equilibrated in equilibration buffer (50 mM Tris-Cl, pH 8.8, 6 M urea, 30% glycerol, 2%SDS, trace of bromophenol blue) containing 10 mg/mL dithiothreitol for 15 min and then in equilibration buffer containing 25 mg/mL iodoacetamide for 15 min. The second dimension was run on 12% acrylamide gels and stained with SYPRO Ruby (Life technologies) before scanning on ProExpress 2D (PerkinElmer). Gels were analysed using the Progenesis Same Spots software (Nonlinear Dynamics/Waters, Durham, NC, USA, version 3.0) using non-linear dynamics. Spots were cut using ProXcision robot (PerkinElmer) and identified using nano LC C18 separation tandem mass spectrometry detection (Proteomics Platform of the Eastern Quebec Genomics Center, Québec, QC, Canada) following trypsin digest as described below. 

### 2.8. Protein In-Gel Digestion and Spot Identification

The three fractions per line of the SDS-PAGE gels as well as protein spots extracted from 2D gels were washed extensively with HPLC water. Proteins were digested in-gel using the MassPrep liquid handling station (Waters, Mississauga, ON, Canada) according to manufacturer’s instructions. Porcine trypsin (Sequencing grade, Promega, Madison, WI, USA) was used to digest proteins at 58 °C for one hour and the products were extracted in 1% formic acid, 2% acetonitrile, followed by 1% formic acid, 50% acetonitrile. Peptides were dried in a speed vacuum and re-suspended in 8 µL 0.1% formic acid. Four µL of re-suspended peptides were separated and ionised using a BioBasic C18 reversed phase column (pore size 300 Å, particle size 5µm) with a PicoFrit 15 µL tip (New Objective, Woburn, MA, USA). An LTQ linear ion trap mass spectrometer, equipped with a nanoelectrospray ion source (Thermo Electron, Waltham, USA) was used to detect the ions. A linear gradient from 2% to 50% acetonitrile in 0.1% formic acid was used at a flow rate of 200 nL/min for a total run time of 42 min. Excalibur software (Thermo Fisher) was used to acquire mass spectra for each full scan mass spectrum followed by collision-induced dissociation spectra for the seven most abundant ions. The dynamic exclusion function was enabled (at 30 s exclusion), and the relative collisional fragmentation energy was set to 35%. MS/MS spectra were analysed using Mascot (Matrix Science, Boston, MA, USA, version 2.2.0) and searched against *Leishmania* in the TriTryp database version 4.0 using trypsin as the protease. A mass tolerance of 2.0 Da for peptides and 0.5 Da for fragments was used, with two trypsin miss cleavages allowed. Carbamidomethylation of cysteine and partial oxidation of methionine modifications were considered in the search. The Scaffold software (Proteome Software, Portland, OR, USA, version 4) was used to validate MS/MS-based peptide and protein identifications. Peptide identifications were accepted if they reached greater than 95% probability and contained at least two unique peptides as specified by the Protein Prophet algorithm. Identifications that were recurrent in many 2D spots in different locations (e.g., α- and β-tubulins) were excluded (unless the peptides matched a section of a larger protein and therefore could represent a fragment of the protein). The relevant mass spectrometry data for the proteins and peptides identified in this study can be found in Supplementary File 2 for the three SDS-PAGE gel slices and in Supplementary Files 3 and 4 for the spots recovered from the 2D gels. 

## 3. Results

### 3.1. Extracting and Analysing Purified Mitochondria

**Figure 1:**
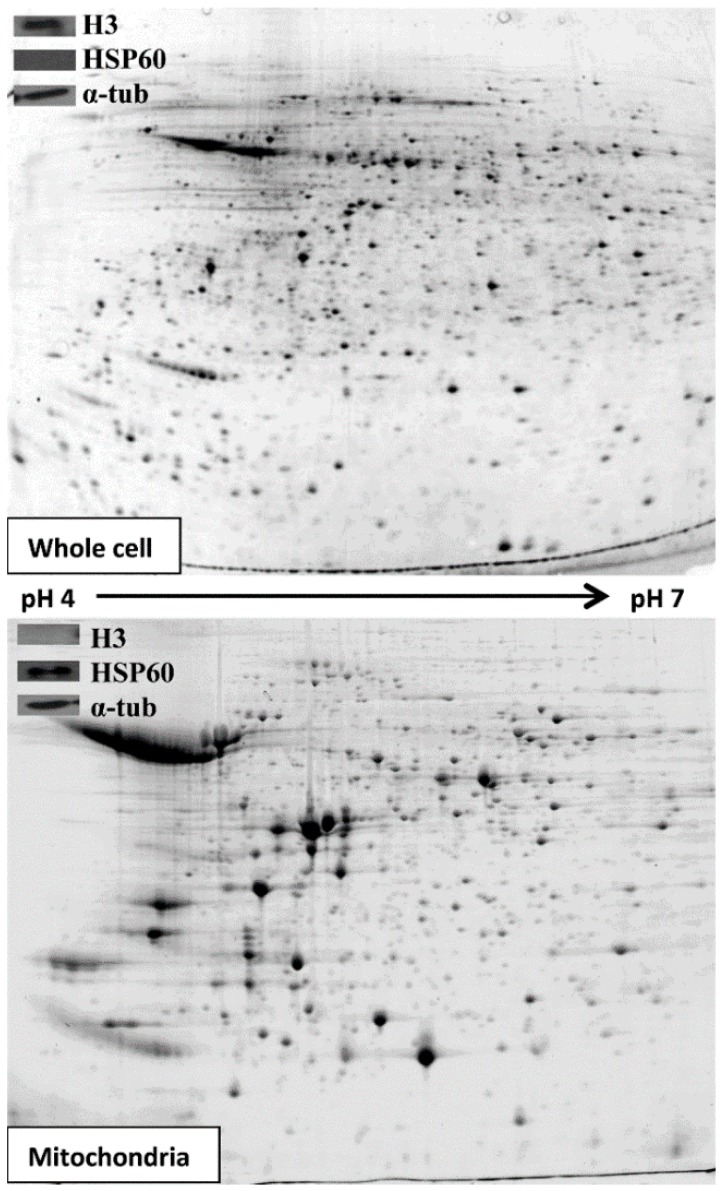
Representative 2D gels of soluble proteins from whole JPCM5 WT cells (top) and extracted mitochondria (bottom). Insets: Western blots of SDS-PAGE from whole JPCM5 WT cells (top) and extracted mitochondria (bottom) showing histone 3 (H3, a nuclear protein), HSP60 (a mitochondrial protein) and α-tubulin (α-tub, a loading control) (15 µg of protein).

The purpose of this work was to carry out proteomics of enriched *Leishmania* mitochondrial fractions and to compare those fractions derived from either sensitive or resistant parasites. To verify that our protein samples were enriched for mitochondria we performed a western blot for HSP60 and to show that they did not contain nuclear contamination, we performed a western blot for histone 3 (a nuclear protein) (Figure 1, insets). The whole cell extract showed signal for histone 3 and therefore indicated the presence of nuclei as a positive control (Figure 1, insert upper panel). The 25%:28% interface of a Nycodenz gradient had no signal for histone 3, showing that there was no, or a greatly reduced level of nuclear proteins, but did show the presence of HSP60, a marker for mitochondrial protein (Figure 1, insert lower panel). When the same amount of protein (200 µg) derived from the 25%:28% fraction or from whole cell extracts was migrated on 2D gels, fewer spots were observed in the mitochondrial samples compared to whole cell extracts (Figure 1), indicating an enrichment of specific proteins in the mitochondrial samples.

To further evaluate the extent of mitochondrial enrichment, the proteins extracted from WT cells present in the 25%:28% Nycodenz interface were separated on SDS-PAGE gels. Each sample line from the WT extracts was cut in three gel pieces, proteins were in-gel digested then sent for mass spectrometry analysis. About 33% of the proteins (116 out of 353 identifications, see Supplementary File 5) appeared to correspond to mitochondrial proteins as deduced from detected mitochondrial targeting sequences using the MitoProt algorithm. The other ~67% contained histones, glycosomal proteins, tubulins, few cytosolic proteins, and hypotheticals with unknown function or proteins not previously reported to be mitochondrial. Some of these proteins, such as the mitochondrial carrier proteins LinJ.02.0640, LinJ.14.1050 and LinJ.30.1110 (Supplementary File 5), were not predicted to be mitochondrially located according to MitoProt but probably did have a mitochondrial location as inferred by similarity against homologous proteins. Moreover, the fact that some proteins are inserted into the membrane (for example the ATP synthase corresponding to complex V in the mitochondrial respiratory chain) or are carried into the mitochondrion by other proteins [30] means that the lack of signalling peptides does not preclude a mitochondrial location. The false positive and false negative rates calculated from MitoProt for the present study (see confidence intervals tab in Supplementary File 5) are similar to the rate values obtained in *T. brucei* [27], validating further our purification scheme for mitochondrial proteins in *Leishmania*. Thus, based on the false positive and false negative rates, we can conclude that the separation of mitochondrial proteins in SDS-PAGE gels allowed us to identify by MS/MS between 92 (the higher confidence limit) to 345 mitochondrial proteins (the lower confidence limit). 

### 3.2. Comparative 2D Gels 

We wanted to compare 2D gels of the wild-type mitochondrial proteome to those of the MF200 and SbIII2000.2 resistant lines. This was achieved using the Progenesis Same Spots software package. Thirty spots were chosen for identification by mass spectrometry (Supplementary File 6). To minimize the possibility of artefacts originating from reproducibility issues between 2D gels replicates, the 30 spots were selected after data filtration based on two factors: a *p*-value of less than 0.05 in a Student’s *t*-test comparing resistant expression to WT and a difference in expression of more than two-fold in at least one of the two resistant mutants. Protein identifications were recovered from 28 spots and these were listed in Table 1. Protein identification by sensitive methods such as LC-MS/MS often reveals more than one protein per spot, in which case the second best protein hits based on the number of unique peptides and coverage were also included in Table 1. One of the protein spots (spot #2601, Supplementary File 6) could not be identified whereas another gave a protein hit for α-tubulin (spot #1969, Supplementary File 6) and was thus discarded because of the recurrence of this protein in several 2D spots (see Experimental Section).

**Table 1:** Identifications of spots with differences in abundance between wild-type and miltefosine resistant or antimony resistant *L. infantum* enriched mitochondria.

Progenesis 2D Spot ^#^	ID ^1^	Accession Number	Probability of Export to Mitochondrion ^2^	Total Spectrum Count	% Sequence Coverage	Fold Change c.f. WT		Molecular Weight ^3^ (KDa)	Isoelectric Oint ^3^	Secondary ID ^4^
						MF200	SbIII 2000.2	Th/Exp	Th/Exp	
687	Dihydrolipoamide dehydrogenase, putative	LinJ.32.3510	0.0841	63	32	0.77	0.36 *	51/65	6.87/6.41	LinJ.33.2570
702	Hypothetical protein, conserved	LinJ.29.0940	0.9761	70	34	2.90 *	1.91 *	53/64	7.8/6.54	LinJ.35.1390
721	Axoneme central apparatus protein, putative	LinJ.20.1450	0.3102	410	68	1.80	5.56 *	55/63	5.72/5.91	LinJ.12.0580 (ALAT)
765	Hypothetical	LinJ.30.3740	0.0665	7	9.5	0.28 *	0.18 *	51/61	5.98/6.13	None
793	Hypothetical protein, conserved	LinJ.29.0940	0.9761	418	43	0.30 *	0.21 *	53/60	7.8/5.96	LinJ.34.3460
921	Dihydrolipoamide dehydrogenase, putative	LinJ.32.3510	0.0841	223	55	0.46 *	0.22 *	51/55	6.87/6.39	LinJ.36.5380
1018	Hypothetical	LinJ.29.0940	0.9761	186	36	0.56 *	0.47 *	53/51	7.8/6.27	LinJ.34.0560
1313	Hypothetical protein containing WD repeats and a STRAP motif	LinJ.27.1140	0.0772	64	60	2.75 *	1.38	35/41	6.18/6.06	LinJ.28.2950
1499	GTP-binding protein, putative, Probable dynamin-1-like protein	LinJ.29.2310	0.0951	8	8.40	0.20 *	0.16 *	78/36	7.49/5.86	LinJ.25.1210
1524	Hypothetical (first half)	LinJ.36.5380	0.9460	168	25	0.25 *	0.16 *	71/35	5.69/5.73	None
1655	Pyruvate dehydrogenase E1 beta subunit, putative	LinJ.25.1790	0.9909	833	52	0.29	0.14 *	38/32	5.72/6.15	None
1664	Calcium binding protein, putative	LinJ.30.1300	0.1494	19	16	0.22 *	0.22 *	59/32	7.42/5.66	LinJ.25.1790
1689	GTP-binding protein (putative)	LinJ.25.1460	0.0197	117	53	2.06	3.09 *	24/31	6.51/6.29	None
1690	Hypothetical (second half)	LinJ.36.5380	0.9460	80	21	2.68 *	1.30	71/30	5.69/5.85	LinJ.30.1920
1757	Hypothetical	LinJ.25.1720	0.9632	22	25	2.51 *	2.52	26/28	8.62/6.29	LinJ.16.1510
1809	Hypothetical	LinJ.36.7070	0.8229	108	36	0.57	0.22 **	29/27	5.56/5.81	None
1855	isocitrate dehydrogenase [NADP], mitochondrial precursor, putative	LinJ.10.0310	0.8889	3	8	0.25	0.12 *	48/25	8.51/6.24	None
1904	Hypothetical	LinJ.25.2520	0.0044	146	7.5	0.37 *	0.40	109/23	7.36/5.15	None
1918	orotidine-5-P decarboxylase/orotate phosphoribosyltransferase, putative	LinJ.16.0560	0.4661	14	14	0.26 *	0.23 **	50/22	9.41/5.62	None
1921	orotidine-5-P decarboxylase/orotate phosphoribosyltransferase, putative	LinJ.16.0560	0.4661	11	15	0.19 *	0.11 **	50/22	9.41/6.00	None
1985	Flavoprotein subunit-like protein	LinJ.07.0910	0.5405	12	17	2.43 *	1.69	61/19	8.84/5.32	LinJ.15.0320
2036	Hypothetical	LinJ.21.1560	0.7471	19	13	0.59 *	0.31 *	39/18	4.94/6.33	None
2042	pyruvate dehydrogenase E1 beta subunit, putative	LinJ.25.1790	0.9909	25	39	0.68	0.38 *	38/17	5.72/5.5	LinJ.22.0900
2108	Hypothetical	LinJ.35.3770	0.0528	12	13	0.27 *	0.20 *	51/<14	7.51/5.12	None
2145	Hypothetical	LinJ.26.1020	0.1396	37	6.9	0.36 *	0.25 *	60/<14	6.87/5.32	LinJ.29.0940
2540	Hypothetical protein containing an EF-hand calcium binding domain	LinJ.34.2780	0.0227	309	60	1.28	2.61 *	47/56	5.35/5.66	None
2605	i/6 autoantigen-like protein	LinJ.22.1310	0.1369	199	59	2.67 *	2.2 *	23/30	5.53/6.08	None
2625	Succinyl-coa:3-ketoacid-coenzyme a transferase like protein	LinJ.30.1920	0.9835	371	49	1.48	3.10 *	53/68	7.15/6.61	LinJ.32.3510

^#^ Spot number. * *p* < 0.05, ** *p* < 0.001. See Supplementary Files 3, 4 and 6 for more details; ^1^ Primary IDs correspond to proteins supported by the higher number of unique peptides and coverage value; ^2^ Predicted using MitoProtII v1.101 [31]. A MitoProt score cut-off of more than 0.5 predicts these proteins to be mitochondrial. Secondary IDs = other potential identifications (second best protein hit) for the spot based on the peptides identified; ^3^ Experimental PIs and MWs were calculated using the Progenesis Same Spots “PI and MW calibration” tool. The theoretical PI values of proteins were found on scaffold software which is linked to TritrypDB database (http://tritrypdb.org/tritrypdb/); ^4^ Secondary IDs correspond to the second best protein hit based on the number of unique peptides and coverage value.

**Figure 2:**
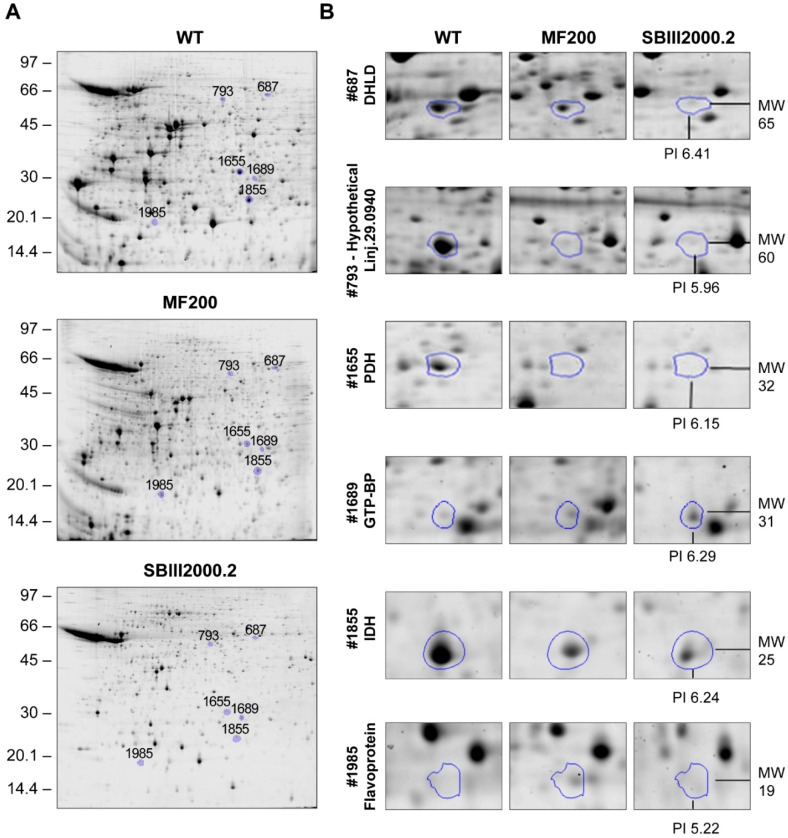
Separation of mitochondrial proteins extracted from JPCM5 wild-type, MF200 and SbIII2000.2 (**A**) Representative 2D gels for JPCM5 wild-type, MF200 and SbIII2000.2; (**B**) Zooms of 2D gels presenting selected protein spots in JPCM5 wild-type, MF200 and SbIII2000.2 2D gels highlighted using Progenesis Same Spots. Spot #687, dihydrolipoamide dehydrogenase (DHLD); Spot #793, hypothetical protein LinJ.29.0940; Spot #1655, pyruvate dehydrogenase E1 β-subunit (PDH); Spot #1689, GTP binding protein (GTP-BP); Spot #1855, isocitrate dehydrogenase (IDH); Spot #1985, Flavoprotein subunit-like protein (Flavoprotein). A representative spot from the four (three for wild-type) independent replicates is depicted.

Thirteen of the identifications were predicted by Mitoprot to be mitochondrial (Table 1), three of which (e.g., the isocitrate dehydrogenase (IDH, spot #1855), pyruvate dehydrogenase (PDH, spots #1655 and #2042) and succinyl-coa:3-ketoacid-coenzyme A transferase like protein named from here on as succinyl transferase (spot #2625)) were previously predicted to be mitochondrial from the analysis of the *T. brucei* mitochondrial proteome [27] further confirming that we have enriched for mitochondrial fractions. Interestingly, the dihydrolipoamide dehydrogenase (DHLD, spots #687 and #921) was not predicted to be mitochondrial by MitoProt but was detected in the mitochondrion in the *T. brucei* screen [27]. Some likely cellular contaminants were also detected in our mitochondrial enriched extracts namely the glycosomal bifunctional enzyme orotidine-5P-decarboxylase/orotate phosphoribosyltransferase (spots #1918 and #1921) involved in the *de novo* pyrimidine biosynthesis, the axoneme central apparatus protein (spot #721) and the i/6 autoantigen-like protein (spot #2605).

**Figure 3:**
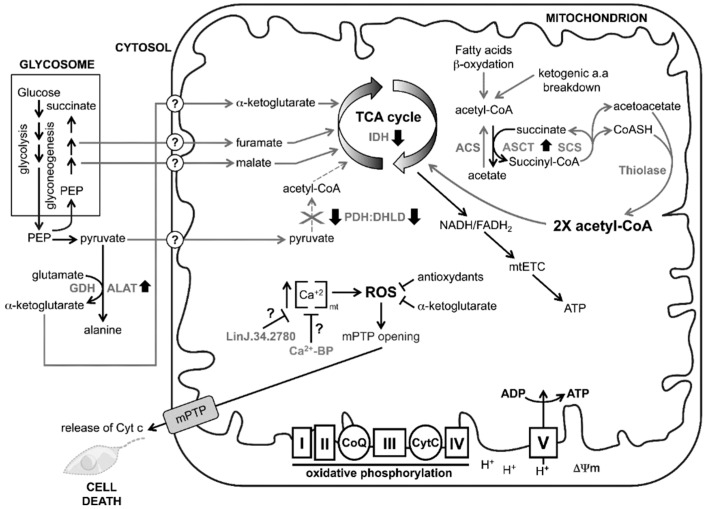
Remodelling of mitochondrial metabolism in miltefosine and antimony resistant mutants to evade cell death. The mitochondrion is at the core of cellular energy metabolism, being the site of most ATP generation through complex V supported by the electron transport chain (mETC). During oxidative phosphorylation, electrons liberated from reducing substrates are delivered to the final acceptor O_2_ via a chain of respiratory hydrogen (H^+^) pumps composing the mtETC (complex I to IV). These pumps establish a H^+^ gradient across the inner mitochondrial membrane, and the electrochemical energy of this gradient is then used to drive ATP synthesis by complex V (e.g., ATP synthase) which promotes the maintenance of the mitochondrial membrane potential (Δψm). The chemical reduction of O_2_ into H_2_O then proceeds via several reactive oxygen species (ROS) produced during the normal oxidative phosphorylation process, which is inevitable, and can damage cellular components such as proteins, lipids, and nucleic acids. The control of ROS production in the single mitochondrion is thus crucial for the organelle integrity and proper function in *Leishmania*. Miltefosine and antimony in susceptible *Leishmania* cause an increase in the ROS production which leads to parasite cell death. In resistant parasites, almost no ROS overproduction is observed which suggests that resistant parasites are able to manage more efficiently ROS upon drug treatment or are able to block or compensate the effects of these drugs on ROS overproduction. This is achieved by a remodelling of mitochondrial metabolism in mitefosine and antimony resistant mutants to evade cell death. Miltefosine and antimony cause a mitochondrial uncoupling process through the down-regulation of the pyruvate dehydrogenase (PDH)/dihydrolipoamide dehydrogenase (DHLD) complex. The PDH/DHLD complex in WT conditions catalyzes the oxidative decarboxylation of pyruvate to form acetyl coenzyme A (acetyl-CoA), the fuel for the citric acid (TCA) cycle. A decrease in the TCA cycle activity would lead to a decrease in the generation of NADH/FADH_2_ and to the uncoupling of the electron transport chain which ultimately leads to cell death. A downregulation of the PDH/DHLD complex may lead the resistant cells to reprogram their intermediary metabolisms to resist drug induced cell death. This could be achieved, in part, by several metabolic pathways highlighted in the present study that are modulated in SbIII and MIL resistant mutants (see discussion for further details). Resistant SbIII and MIL resistant *Leishmania* mutants are able to maintain Ca^2+^ and ROS homeostasis within the organelle, escaping cell death pathways. IDH, isocitrate dehydrogenase; ALAT, alanine aminotransferase; ACS, acetyl-CoA synthetase [32]; ASCT, acetate:succinate CoA transferase also known as succinyl transferase enzyme; GDH, glutamate dehydrogenase; PDH, pyruvate dehydrogenase; DHLD, dihydrolipoamide dehydrogenase; CoQ, coenzyme Q; Cyt c, cytochrome c; TCA cycle, tricarboxylic cycle; PEP, phosphoenolpyruvate; “?”, unknown mitochondrial transporter for malate, furamate, α-ketoglutarate and pyruvate.

A sample of these spots having an altered abundance in both mutants compared to WT mitochondrial extracts are shown in Figure 2. Interestingly, these spots showed similar expression levels in both mutants (Table 1), suggesting some shared responses in the resistance to either miltefosine or SbIII (Figure 3). A number of the proteins identified as primary hits (Table 1) were known to be involved in the TCA cycle (e.g., DHLD, spots #687 and #921; IDH, spot #1855; and PDH, spots #1655 and #2042), in acetate production (succinyl transferase, spot #2625), and possibly in mitochondrial oxidative respiration (flavoprotein subunit-like protein, spot #1985) and calcium homeostasis (calcium binding protein, spot #1664; hypothetical protein containing an EF-hand calcium binding motif, spot #2540). A number of secondary hits (e.g., secondary IDs in Table 1) were also consistent with the mitochondrial oxidative phosphorylation process e.g., LinJ.25.1210 (spot #1499) encoding an ATPase β-subunit as well as LinJ.34.3460 (spot #793) encoding the vacuolar ATP synthase subunit A. Interestingly, numerous hypothetical proteins of unknown function had an altered abundance in both mutants (Table 1). Altogether, these results suggest that in order to survive the cytotoxic mode of action of miltefosine and antimony, *Leishmania* needs to remodel its mitochondrial metabolism and several proteins identified in the present study were similarly modulated in parasites resistant to each of these drugs. A shared adaptation of the single mitochondrion to these two chemically unrelated drugs was highlighted.

## 4. Discussion

In this study we tested whether SbIII and MIL resistance induced any changes to the mitochondrial proteome, if some of these changes were related to cell death pathways, and whether these changes were common in both mutants. It is worth mentioning that our proteomic approach would reveal differential abundance of soluble proteins but less easily point mutations or modulation in transmembrane proteins. Also of interest, we chose to compare mitochondrial protein extracts derived from parasites in their logarithmic phase because of (i) extensive proteolysis reported in the stationary phase of the parasite [33]; and (ii) more changes are induced by drugs and oxidants, at least in bacteria, in logarithmic phase rather than stationary cells [34]. 

Our mitochondrial protein extracts were enriched for mitochondrial proteins as demonstrated by Western blot hybridizations for nuclear and mitochondrial markers as well as by the identification of proteins derived from a WT mitochondrial extract (Supplementary File 5). Indeed, an important proportion of identified proteins in this extract were either already known to be mitochondrially located or should be targeted to the mitochondrion according to their MitoProt score. However, the WT mitochondria-enriched sample contained some contamination from nuclear, glycosome, and cytosolic proteins (Supplementary File 5). The extent of mitochondrial enrichment was sufficient to proceed with 2D gels/MS for comparative proteomic screening contrasting the MIL and SbIII sensitive and resistant mitochondrial extracts.

Interestingly, 2D gels revealed that some protein spots found in the mitochondrial enriched fractions differ significantly between sensitive and resistant cells but there were also striking similarities pertaining to oxidative processes between the mitochondrial protein profiles from the SbIII and MIL resistant mutants (Table 1), suggesting a shared adaptation to these two chemically unrelated drugs. The single mitochondrion is one of the major sources of ROS in trypanosomatids through the activity of the mitochondrial electron transport chain (mtETC, depicted as complex I to IV in Figure 3) [35] and SbIII and MIL are known to induce cell death in *Leishmania* by producing ROS within the mitochondrion in drug-sensitive parasites. A decrease in energy production results in opening of the mitochondrial permeability transition pore (mPTP), which causes collapse of mitochondrial membrane potential (Δψm), mitochondrial membrane swelling, and indirectly enhances the release of death factors (e.g., cytochrome c (Cytc)) into the cytosol, leading to cell death [25,36] (Figure 3). It was demonstrated that SbIII or MIL resistant parasites present significantly lower levels of ROS compared to wild-type cells [13] and this suggests that SbIII and MIL resistant *Leishmania* are probably able to maintain mitochondrial ROS to a level similar or lower than wild-type, either by more effective scavenging or by decreased ROS production. An increase in the level of several antioxidant molecules has already been reported to help dealing with ROS induced upon drug treatment, including an increase in the levels of glutathione/trypanothione [37,38] and an increase in the activity of the superoxide dismutase A (SODA) [25,39]. Our proteomic work led to the identification of additional factors that may help to control the ROS levels in resistant parasites. Calcium (Ca^2+^) is a well-known key regulator of mitochondrial functions and Ca^2+^ alterations are known to induce ROS production. Calcium homeostasis is crucial for the proper functioning of mitochondria and many antileishmanial agents exert their cytotoxic effects through the disruption of Ca^2+^ homeostasis in the parasite [40,41,42,43]. SbIII and MIL drug treatment may cause mitochondrial matrix Ca^2+^ overload, which can lead to enhanced generation of ROS (Figure 3). We have detected two calcium-binding proteins (spot #1664 and spot #2540) that were respectively down- and upregulated in both the SbIII and MIL resistant mutants (Table 1). Although not predicted to be mitochondrially located by MitoProt (respective scores of 0.1494 and 0.0227), the investigation of their putative role in maintaining mitochondrial Ca^2+^ homeostasis in *Leishmania* in relation to drug resistance is worth pursuing, especially since cross-talk between various calcium storage compartments and mitochondrion exits in parasites [44,45].

A second strategy potentially deployed by the resistant parasites to better scavenge ROS is the apparent overproduction of alanine aminotransferase (ALAT) (Figure 3). While not a mitochondrial protein per se, ALAT has been detected in both mutants as a secondary hit in our proteomic screen (spot #721, Table 1), and its apparent overexpression may suggest increased alanine and α-ketoglutarate production through the activity of glutamate dehydrogenase (GDH, Figure 3). α-ketoglutarate has been shown to be a key participant in the detoxification of ROS in several organisms [46]. Since isocitrate dehydrogenase (IDH, Figure 3) is downregulated in both MIL and SbIII mutants (spot #1855, Table 1 and see discussion below), which normally produces α-ketoglutarate from isocitrate in the TCA cycle, the upregulation of ALAT, whose activity is synchronized with GDH, may bypass the IDH downregulation observed in the two mutants. This α-ketoglutarate synthesized in the cytosol could then enter the mitochondrion either to sustain the TCA cycle or be used as an antioxidant against ROS. 

Our proteomic comparative analysis revealed the downregulation in both mutants of three key enzymes related to the TCA cycle, namely the pyruvate dehydrogenase (PDH, spot #1655), the dihydrolipoamide dehydrogenase (DHLD, spots #687 and #921), both forming a functional PDH:DHLD complex involved in the conversion of pyruvate to acetyl-CoA, and as indicated above, the isocitrate dehydrogenase (IDH, spot #1855) (Table 1 and Figure 3). The downregulation of these three enzymes suggests that the activity of the TCA cycle in both mutants is diminished compared to WT cells. A decrease in TCA cycle activity would lead to a decrease in the generation of NADH/FADH_2_ and to the uncoupling of the mtETC that should lead to cell death (Figure 3). To resist to the cytotoxic mode of action of MIL and SbIII, resistant parasites need to maintain the integrity of the mtETC as well as their basal ATP production through complex V. The downregulation of the PDH:DHLD complex could cause an apparent shortage in acetyl-CoA, the main fuel of the TCA cycle. If acetyl-CoA cannot be obtained from pyruvate in resistant parasites, it must come from other routes. The two alternative pathways used to generate acetyl-COA in most cell types are the fatty acid β-oxidation pathway and the breakdown of ketogenic amino acids (Figure 3). It is salient to point out that both resistant parasites overproduce the succinyl transferase enzyme (also known as the acetate:succinate CoA transferase, ASCT (spot #2625)) which produces acetate and succinyl-CoA [47] (Figure 3). The succinyl-CoA that is formed as a result of the ASCT reaction could subsequently be converted back into succinate by the succinyl-CoA synthetase (SCS), in a reaction generating coenzyme A (CoASH) and acetoacetate. This acetoacetate could form two molecules of acetyl-CoA which could enter the TCA cycle (Figure 3). The ASCT-SCS cycle part of the PDH:DHLD bypass pathway has been identified in trypanosomatids [48]. Further investigations are required to confirm the presence of this PDH:DHLD bypass pathway in *Leishmania* and to identify the putative thiolase but it is noteworthy that three different thiolases are encoded in the genome of *Leishmania* with at least one in the mitochondrion [49]. Alternatively, an acetyl-COA synthase (ACS in Figure 3) recently described in *Leishmania* [32] might be used to reconvert acetate to acetyl-CoA.

Another protein identified is the dynamin-1-like protein (spot #1499) that was down-regulated in both the MIL and SbIII resistant mutants (Table 1). Dynamins are large guanosine triphosphatases (GTPase) that are, amongst other functions, of the mitochondrial fission machinery and have been universally conserved throughout evolution. At least three family members are present in most eukaryotes but only one family has been described in *Trypanosoma brucei* and *Leishmania* *spp.* [50]. In *T. brucei*, the single dynamin-1-like protein (TbDLP) functions in the regulation of mitochondrial membrane division [50,51]. The dynamin-1-like protein LinJ.29.2310 may have similar roles in *Leishmania*. A decrease in the unique dynamin-1-like protein in *Leishmania* may impact the function and morphology of the mitochondrion in resistant mutants and its downregulation may also explain the net reduction in growth rate observed in both resistant cells (Supplementary File 1, Figure S1) as it was reported for *T. brucei* in TbDLP RNAi experiments [50]. 

## 5. Conclusions

We have carried out here the first proteome analysis of the *Leishmania* mitochondrion. We have shown that several proteins enriched in mitochondrial fractions had a differential abundance in both SbIII and MIL resistant *Leishmania*. Since both drugs induce ROS accumulation in sensitive cells but not in resistant cells [13], it is possible that some of the highlighted proteins contribute to this phenomenon which appears central to both mode of action and resistance mechanisms.

## Supplementary Materials

Supplementary File 1
