# Supplementary material for: Mitochondrial Proteomics of Antimony and Miltefosine Resistant Leishmania infantum
Source: Proteomes. 2015 Oct 21;3(4):328–46. doi: 10.3390/proteomes3040328 (PMC5217391; doi:10.3390/proteomes3040328)
Supplement: Supplementary File 1 [file proteomes-03-00328-s001.zip › proteomes-03-00328-supplementary/Supplementary File 1.pdf]

Supplementary Materials

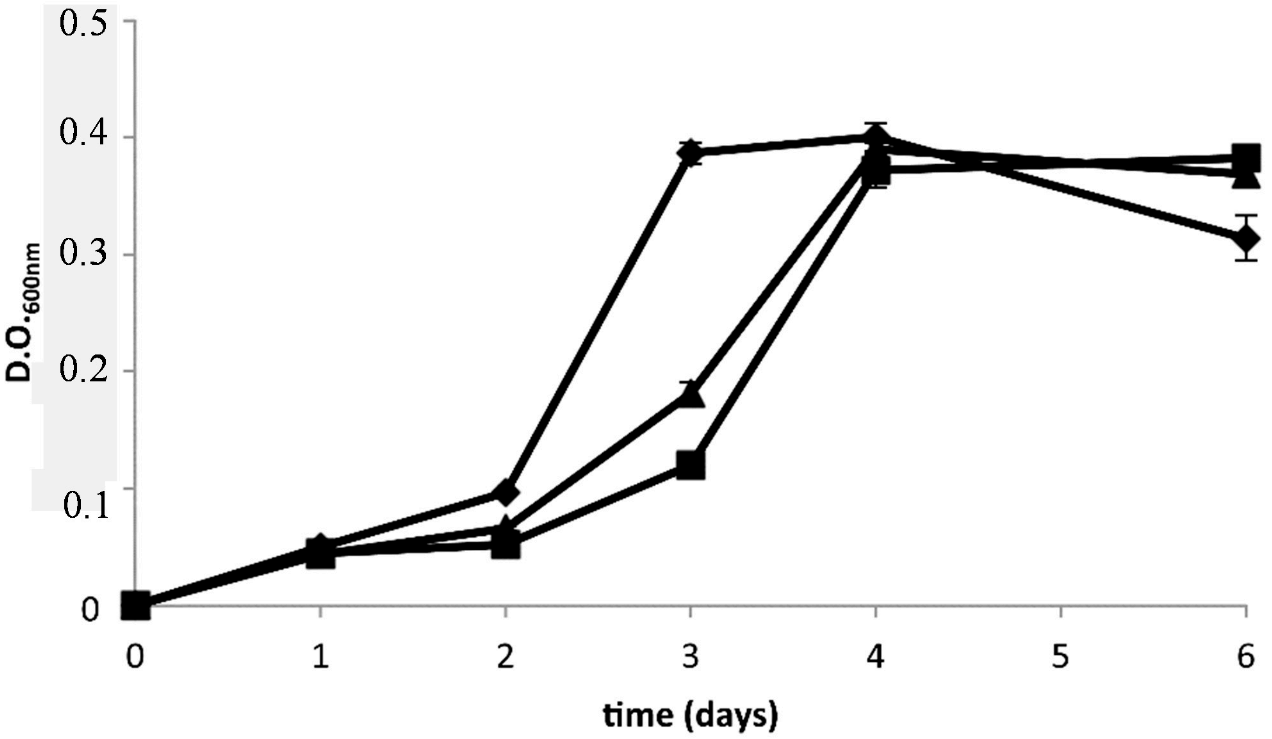

Figure S1. Growth curves for wild-type, MF200 and SbIII2000.2 cell lines. Cell growth was monitored by measuring the absorbance of culture aliquots at 600_{nm} in a multiwell scanning spectrophotometer. JPCM5 wild-type strain (♦); JPCM5 miltefosine resistant mutant MF200, clone 3 (■); JPCM5 SbIII resistant mutant Sb2000.2, clone 3 (▲). Growth curves were performed in triplicate.

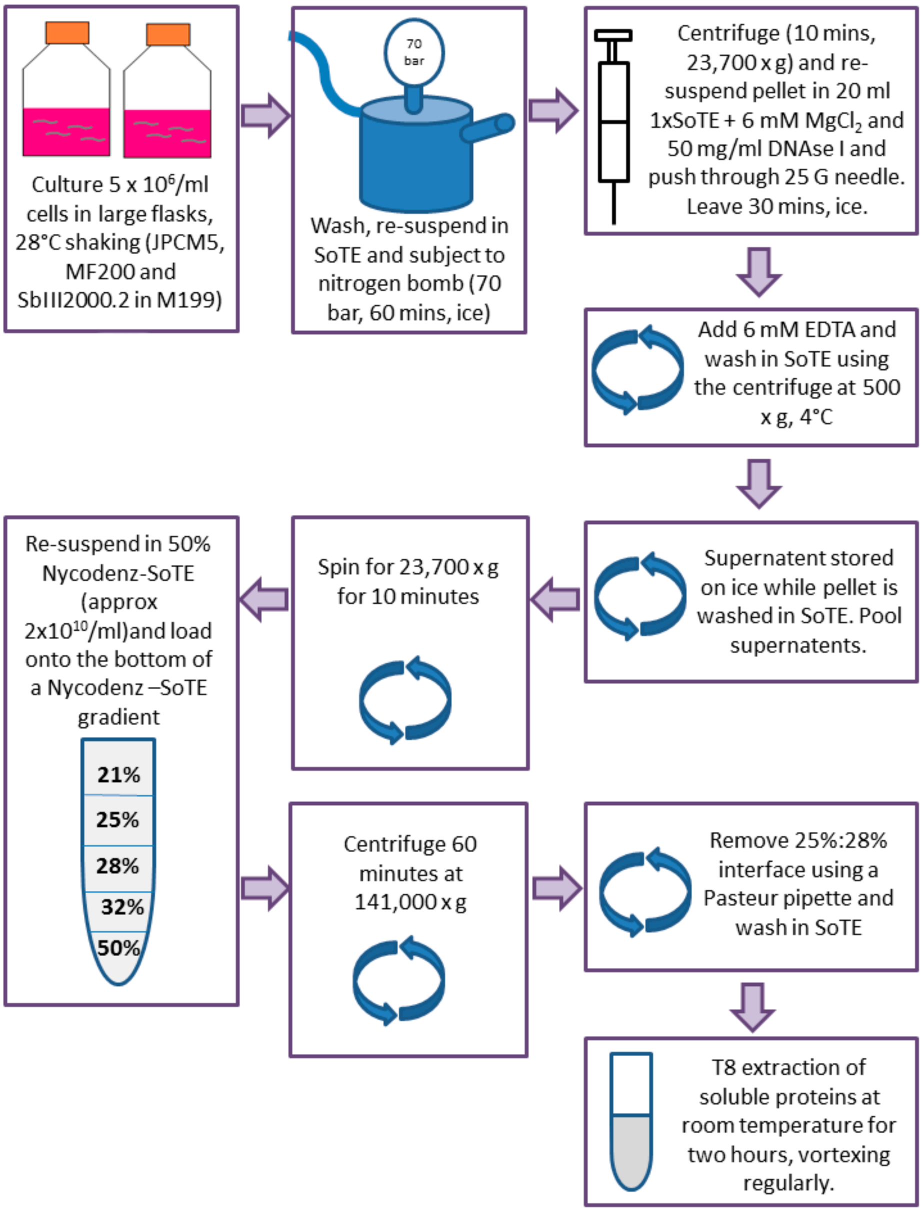

Figure S2. Mitochondrial enrichment procedure. The main steps including buffers and conditions to reproduce this experiment were included.
